# Freezing of Gait in Parkinson's Disease: Don't Leave Patients in the Dark!

**DOI:** 10.1002/mdc3.70401

**Published:** 2025-10-11

**Authors:** Franka Goossens, Gijs Vissers, Bastiaan R. Bloem, Sirwan Darweesh, Jorik Nonnekes

**Affiliations:** ^1^ Department of Rehabilitation, Donders Institute for Brain, Cognition and Behaviour Radboud University Medical Centre Nijmegen The Netherlands; ^2^ Department of Neurology, Donders Institute for Brain, Cognition and Behaviour Radboud University Medical Centre Nijmegen The Netherlands

**Keywords:** freezing of gait, Parkinson's disease, darkness

Freezing of gait (FOG) is among the most burdensome symptoms of Parkinson's disease (PD) according to both patients and caregivers. Patients frequently describe FOG as the feeling as if their feet are “glued” to the floor. Clinically, FOG is defined by a brief, episodic absence or severe reduction in forward movement of the feet despite the intention to walk.^1^ Due to its paroxysmal nature, FOG is a major contributor to falls and fall‐related injuries in people with PD. FOG is typically seen in specific contexts, such as when turning, dual‐tasking, walking under time pressure, and when walking through narrow spaces or doorways. In this video‐illustrated report, we highlight a lesser‐known but clinically relevant context: walking in poorly‐lit environments.

An 80‐year‐old man with a 10‐year history of PD spontaneously reported a worsening of FOG when walking in poorly lit environments. When observed at home, he exhibited FOG during gait initiation and turning even when walking in his well‐lit living room, spending 40% of the time frozen. However, his FOG markedly increased when walking the same trajectory in the dark, spending 72% of the time frozen (Video 1). Interestingly, in both conditions he carried a cane with him, but only in the dark he put it on the ground and used it effectively as an aid, whereas in the light the cane never touched the floor.

**Video 1:** Video of an 80‐year‐old man with a 10‐year history of Parkinson's in his home‐setting, showing a marked increase in freezing of gait when walking in the dark compared to walking along the same trajectory under well‐lit circumstances. Freezing occurs during gait initiation and particularly during turning, well‐known triggers for freezing.

Two mechanisms may be involved in the exacerbation of FOG when walking in the dark, both related to compensation for corticostriatal dysfunction. In PD, gait control depends strongly on compensatory mechanisms, involving cognitive, sensory (e.g., visual), and limbic input. When walking in the dark, there is reduced sensory input, potentially leading to an increase in FOG.^2^ Furthermore, anxiety can increase FOG.^3^ The man highlighted here reported being more anxious when walking in the dark (8 points on a 10‐point visual analogue scale) compared to walking in well‐lit environments (4/10). The noradrenergic ascending arousal system is most likely involved in the integration of compensatory brain networks.^4^ Anxiety can result in heightened arousal, which may over‐enhance the integration between competing inputs of compensatory brain networks, leading to suboptimal compensation.

Interestingly, the fact that the patient did not use the cane effectively when walking in the light may also hint towards anxiety impacting gait control. It has been reported that gait in patients with PD improves in the presence of visual cues, while some persons actually do not look at the presented stimuli.^5^ Most likely, in these patients, the cues act as a fallback option, lowering anxiety and thereby improving gait. In the present case, the walking aid may also have functioned as a fallback option and was used only when needed most: in the dark. The cane may, furthermore, have provided additional sensory input when walking in the dark.

The clinical implication is straightforward: the simple act of turning on lights can reduce FOG severity. Interventions that ascertain adequate lighting conditions, like pathway illumination devices or motion‐activated lights, may lessen the frequency and severity of FOG in low light conditions. Patients must be educated about this to prevent nighttime falls.

## Author Roles

(1) Research project: A. Conception, B. Organization, C. Execution; (2) Statistical Analysis: A. Design, B. Execution, C. Review and Critique; (3) Manuscript Preparation: A. Writing of the first draft, B. Review and Critique.

F.G.: 1B, 1C, 3A.

G.V.: 1B, 1C.

B.B.: 3B.

S.D.: 1A, 3B.

J.N.: 1A, 3A.

## Disclosures

**Ethical Compliance Statement:** This case report was not assesed by an ethical committee but it was conducted in accordance with guidelines of the medical ethical committee Arnhem/Nijmegen. The man with Parkinson's disease and the spotter in the video have signed a consent‐to‐disclose form. We confirm that we have read the Journal's position on issues involved in ethical publication and affirm that this work is consistent with those guidelines.

**Funding Sources and Conflicts of Interest:** No specific funding was received for this work. The authors declare that there are no conflicts of interest relevant to this work.

**Financial Disclosures for the Previous 12 Months:** FG declares that there are no additional disclosures to report. GV declares that there are no additional disclosures to report. BRB serves as the co‐Editor in Chief for the *Journal of Parkinson's Disease*, serves on the editorial board of *Practical Neurology and Digital Biomarkers*, has received fees from serving on the scientific advisory board for the Critical Path Institute, Gyenno Science, MedRhythms, UCB, Kyowa Kirin and Zambon (paid to the Institute), has received fees for speaking at conferences from AbbVie, Bial, Biogen, GE Healthcare, Oruen, Roche, UCB and Zambon (paid to the Institute), and has received research support from Biogen, Cure Parkinson's, Davis Phinney Foundation, Edmond J. Safra Foundation, Fred Foundation, Gatsby Foundation, Hersenstichting Nederland, Horizon 2020, IRLAB Therapeutics, Maag Lever Darm Stichting, Michael J. Fox Foundation, Ministry of Agriculture, Ministry of Economic Affairs & Climate Policy, Ministry of Health, Welfare and Sport, Netherlands Organization for Scientific Research (ZonMw), Not Impossible, Parkinson Vereniging, Parkinson's Foundation, Parkinson's UK, Stichting Alkemade‐Keuls, Stichting Parkinson NL, Stichting Woelse Waard, Health Holland/Topsector Life Sciences and Health, UCB, Verily Life Sciences, Roche and Zambon. BRB does not hold any stocks or stock options with any companies that are connected to Parkinson's disease or to any of his clinical or research activities.

**GRANTS/RESEARCH SUPPORT (paid to the Institute):**

- Cure Parkinson's
- Davis Phinney Foundation
- Edmond J. Safra Foundation
- Fred Foundation
- Gatsby Foundation
- Hersenstichting Nederland
- Horizon 2020
- IRLAB Therapeutics
- Maag Lever Darm Stichting
- Michael J. Fox Foundation
- Ministry of Agriculture
- Ministry of Economic Affairs & Climate Policy
- Ministry of Health, Welfare and Sport
- Netherlands Organization for Scientific Research (ZonMw)
- Not Impossible
- Parkinson Vereniging
- Parkinson's Foundation
- Parkinson's UK
- Stichting Alkemade‐Keuls
- Stichting Parkinson NL
- Stichting Woelse Waard
- Topsector Life Sciences and Health
- UCB
- Verily Life Sciences
- Roche
- Zambon

**CONSULTANT (paid to the Institute):**

- Critical Path Institute
- Gyenno Science
- Remepy
- UCB
- Zambon

**SPEAKING (paid to the Institute)**

- AbbVie
- Bial
- Biogen
- GE Healthcare
- Oruen
- Roche
- UCB
- Zambon

**Stock Shareholder:** None.

**Memberships:**

- Movement Disorder Society
- Co‐Editor in Chief, Journal of Parkinson's Disease
- Member of the Editorial Board, Digital Biomarkers
- Member of the editorial board, Practical Neurology Member Critical Path Institute for Parkinson (https://c-path.org/programs/cpp/)
- Member of the KNAW Van Leersum Fund Review Committee

SD currently serves on the editorial board of *Neurology*, *Frontiers of Neurology* and *Brain Sciences*, has received fees for speaking at conferences and podcasts from AbbVie, and has received research support from the Parkinson's Foundation (PF‐FBS‐2026), ZonMW (09150162010183), ParkinsonNL (P2022‐07 and P2021‐14), Michael J. Fox Foundation (MJFF‐022767), Davis Phinney Foundation and Edmond J. Safra Foundation (all paid to the institute). JN reports grants from ZonMW, Michael J. Fox Foundation, Gossweiler Foundation and ERN‐RND outside the submitted work. He serves on the medical advisory board of Cue2Walk.

## Data Availability

Data sharing not applicable to this article as no datasets were generated or analysed during the current study.
